# Supplementary material for: Reconfigurable, Stretchable Strain Sensor with the Localized Controlling of Substrate Modulus by Two-Phase Liquid Metal Cells
Source: Nanomaterials (Basel). 2022 Mar 7;12(5):882. doi: 10.3390/nano12050882 (PMC8912465; doi:10.3390/nano12050882)
Supplement: Supplementary file 1 [file nanomaterials-12-00882-s001.zip › nanomaterials-1606345-supplementary.pdf]

nanomaterials

Supplementary Materials

Reconfigurable, Stretchable Strain Sensor with the Localized Controlling of Substrate Modulus by Two-Phase Liquid Metal Cells

Linna Mao [1], Taisong Pan [1,*], Junxiong Guo [2], Yizhen Ke [3], Jia Zhu [4], Huanyu Cheng [4] and Yuan Lin [1,5,*]

[1] School of Material and Energy, University of Electronic Science and Technology of China, Chengdu 610054, China; linna_M00@outlook.com
[2] School of Electronic Information and Electrical Engineering, Chengdu University, Chengdu 610106, China; guojunxiong@cdu.edu.cn
[3] School of Electronic Science and Engineering, University of Electronic Science and Technology of China, Chengdu 610054, China; xysfkyz@126.com
[4] Department of Engineering Science and Mechanics, The Pennsylvania State University, University Park, PA 16802, USA; jmz5364@psu.edu (J.Z.); Huanyu.cheng@psu.edu (H.C.)
[5] Medico-Engineering Cooperation on Applied Medicine Research Center, University of Electronics Science and Technology of China, Chengdu 610054, China
* Correspondence: tspan@uestc.edu.cn (T.P.); linyuan@uestc.edu.cn (Y.L.)

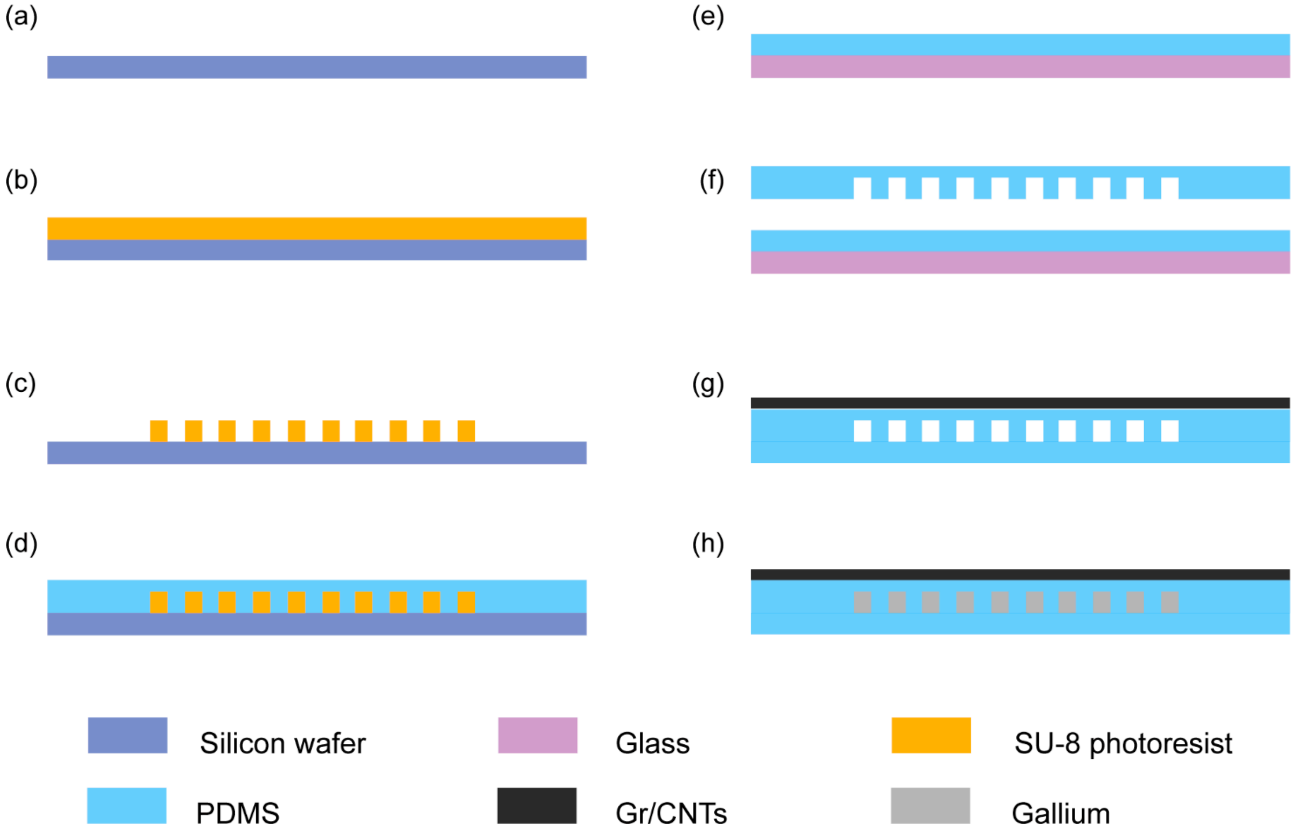

Figure S1. Fabrication process of heterogeneous substrate-based stretchable strain sensor from the view of cross-section. (**a**) Silicon wafer is prepared by deionized water, acetone and plasma treatment; (**b**) SU-8 photoresist was deposited on the silicon wafer by spinning coating method; (**c**) The mold of microchannel was developed by mask lithography; (**d**) The pre-prepared PDMS elastomer is poured into the mold; (**e**) A glass slide was prepared and spin-coated a thinner layer of PDMS mixture; (**f**) The microchannel layer is peeled off from the mold and bonded onto the thinner PDMS layer via wet-bonding. (**g**) The sensing layer which composed of graphene and carbon nanotubes is deposited on the surface by spray coating. (**h**) LM is injected via syringe.

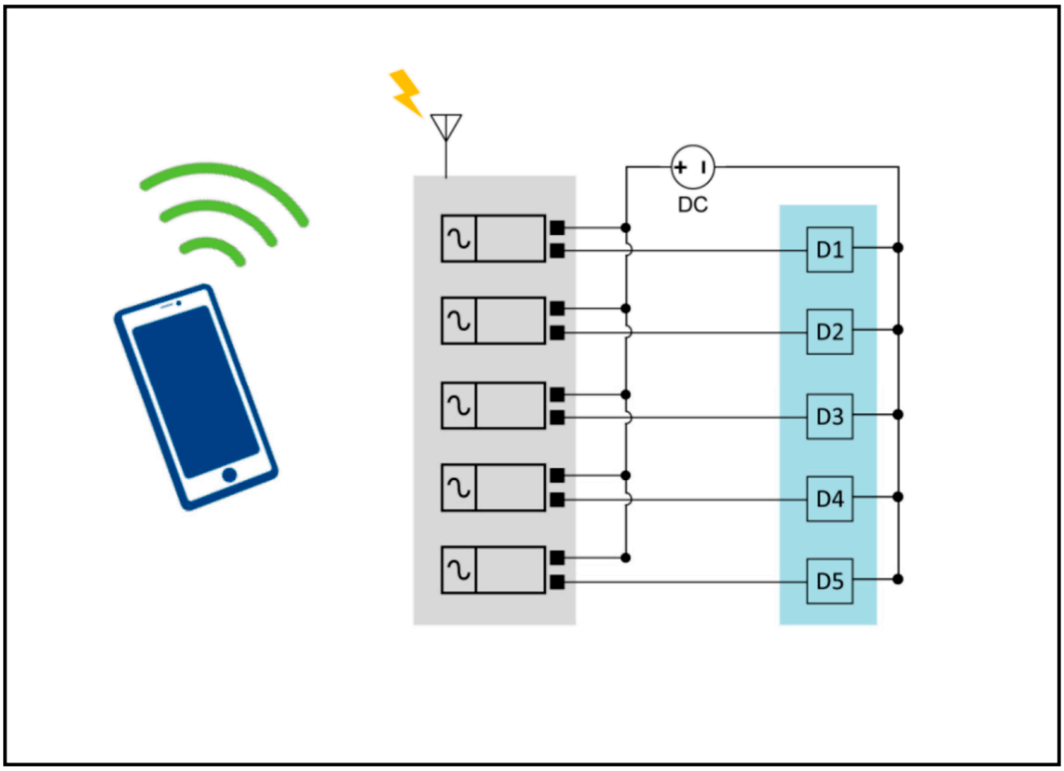

Figure S2. The schematic diagram of the electric heating system.

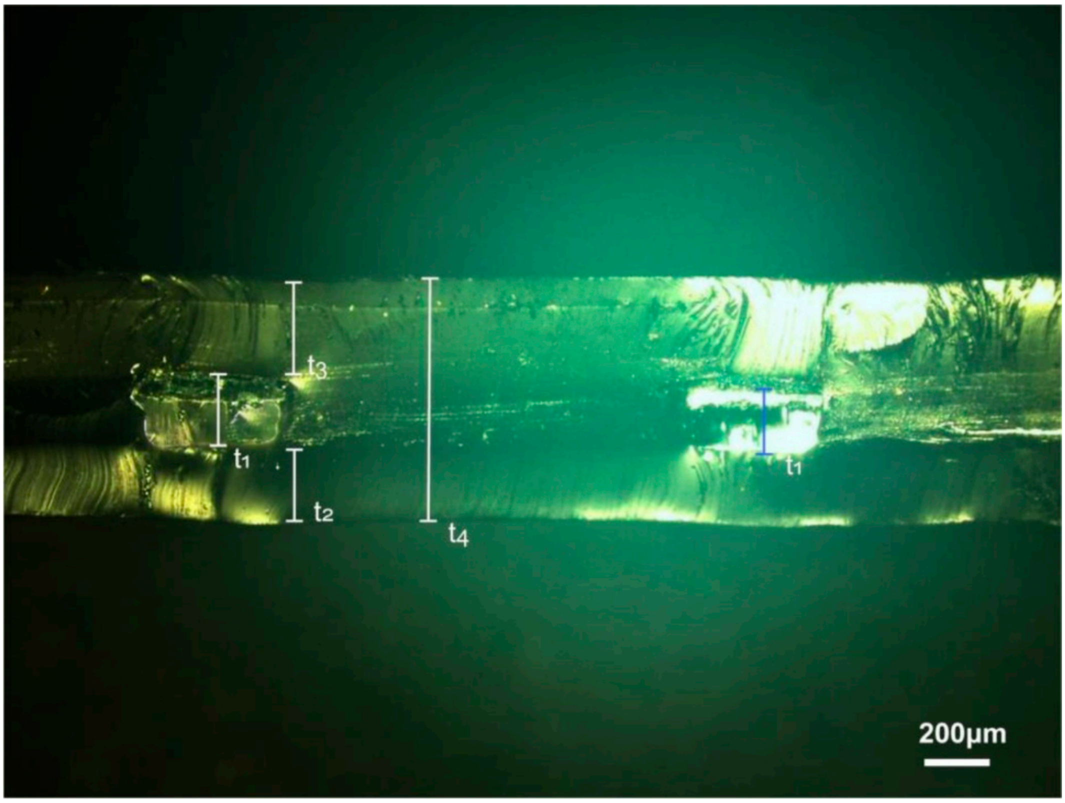

Figure S3. The image of the cross-section of the sensor. The thickness of each layer is as follows: cell layer (t_1): 208 μm, bottom elastomer layer(t_2): 230 μm, upper elastomer layer (t_3): 260 μm, total thickness (t_4): 698 μm.

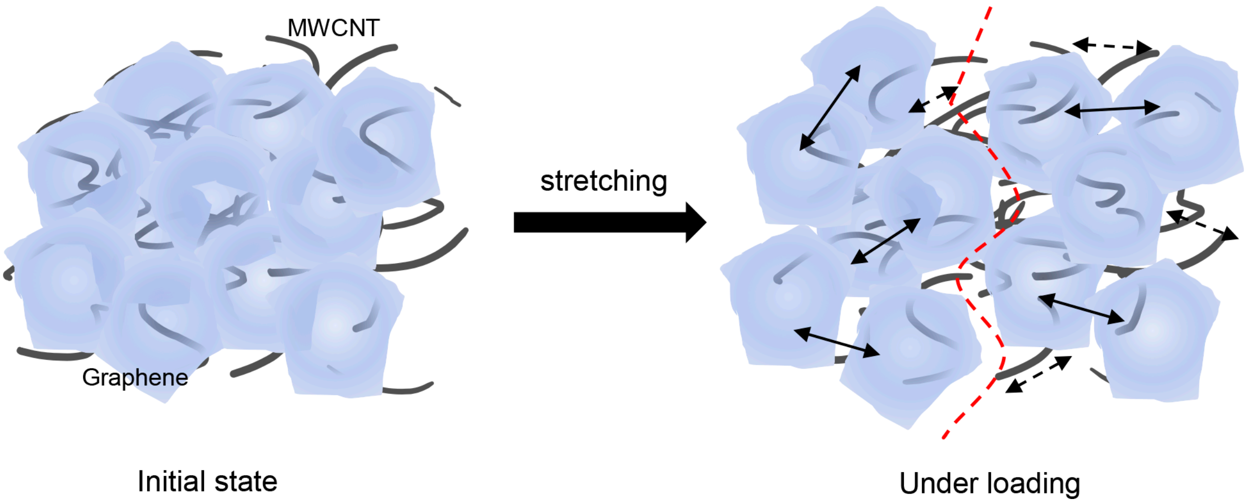

Figure S4. Schematic of the formation of cracks along the boundaries of graphene flakes of the Gr/CNT composite after stretching. Cracks are formed after stretching on the Gr/CNT film surface, especially along with the connection between graphene flakes and the MWCNTs.

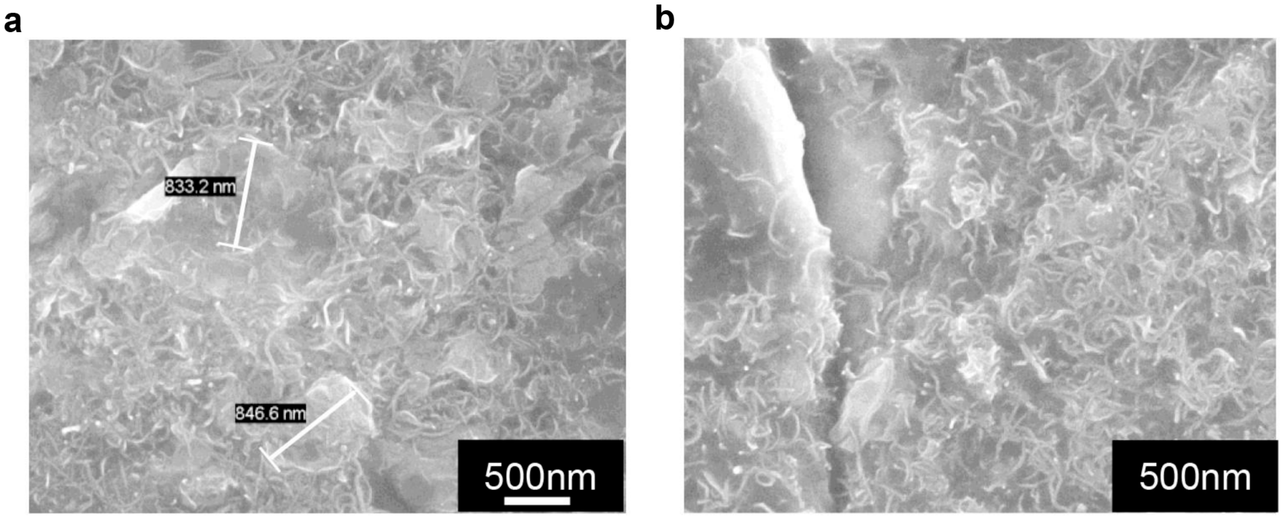

Figure S5. The SEM images of the cross-stacked Gr/CNT hybrid film on the heterogenous PDMS substrate in the view of the top. Scale bar: 500 nm. (**a**) The initial state before a tensile test; (**b**) Return to the initial state after multiple cyclic tensile tests.

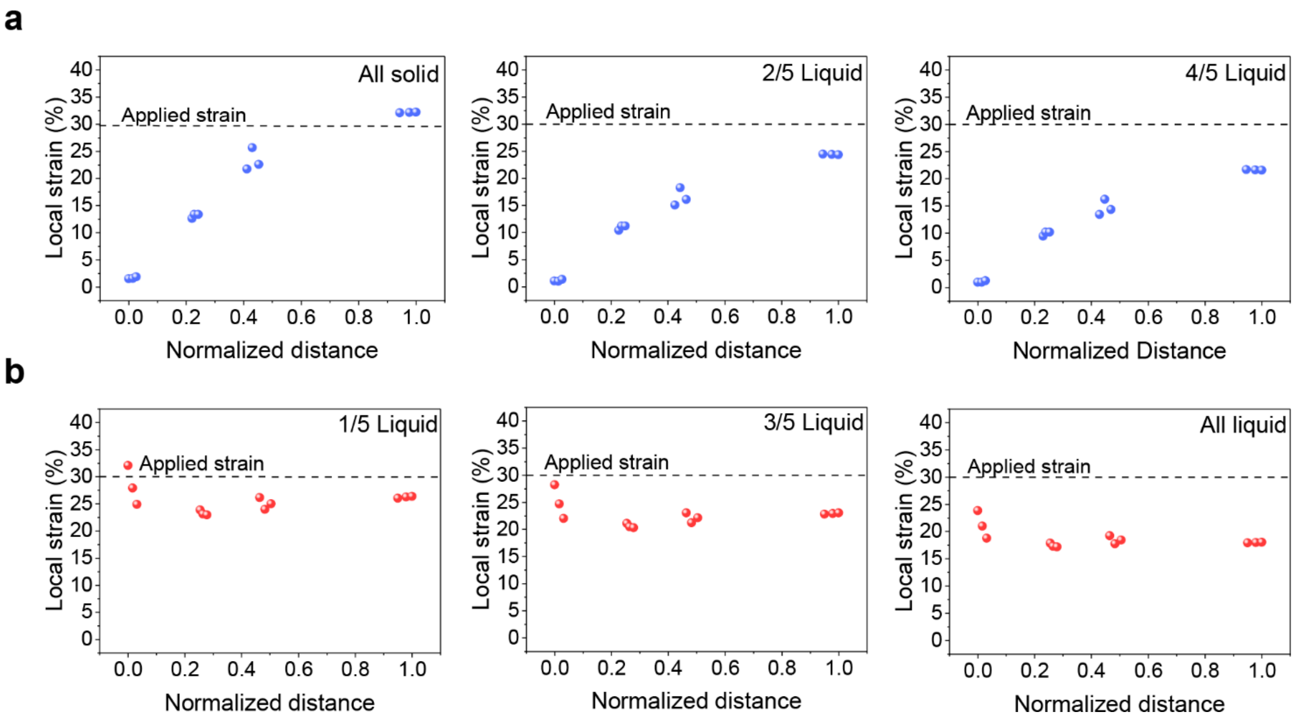

Figure S6. Strain distribution in the substrate along the picked path (shown in Figure 3a) with the different number of "liquid cell" in the substrate. (**a**) the cell at the center of the substrate is in solid state; (**b**) the cell at the center of the substrate is in liquid state.

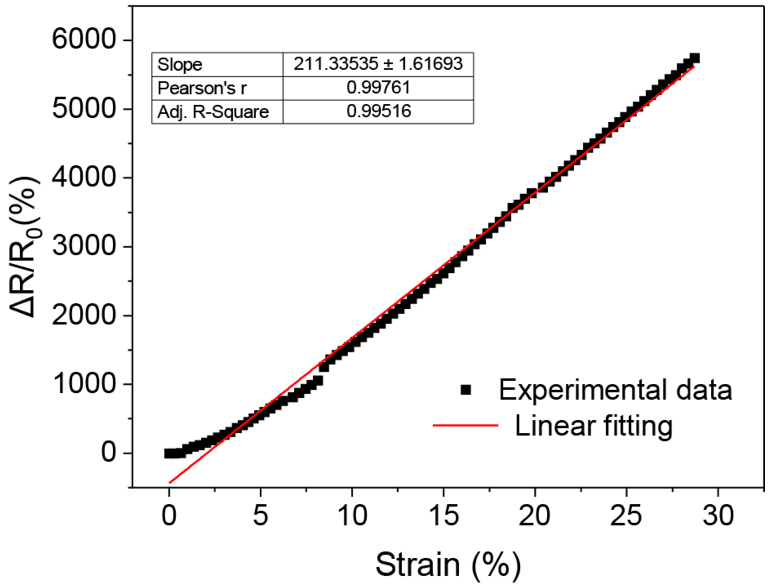

Figure S7. The relative resistance changes along with the applied strain.

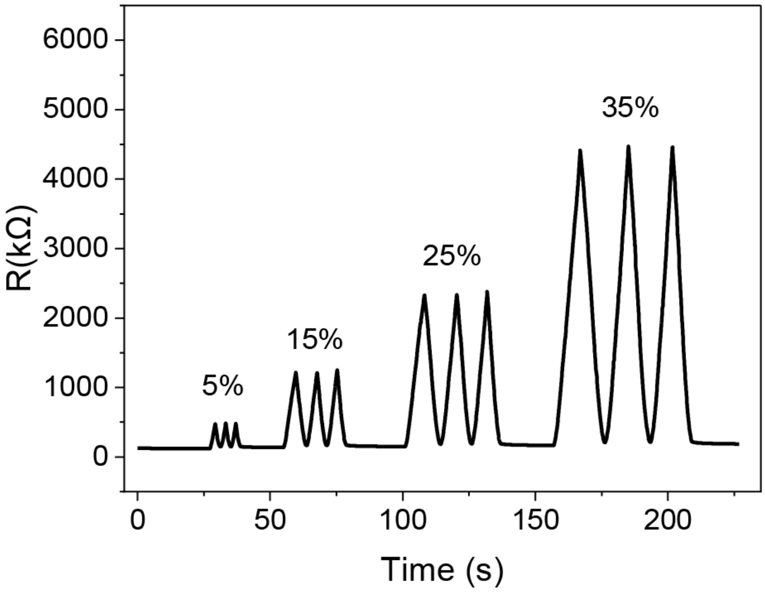

Figure S8. Relative resistance changes during cyclic stretching under difference maximum strains of 5%, 15%,25%,35%, respectively.

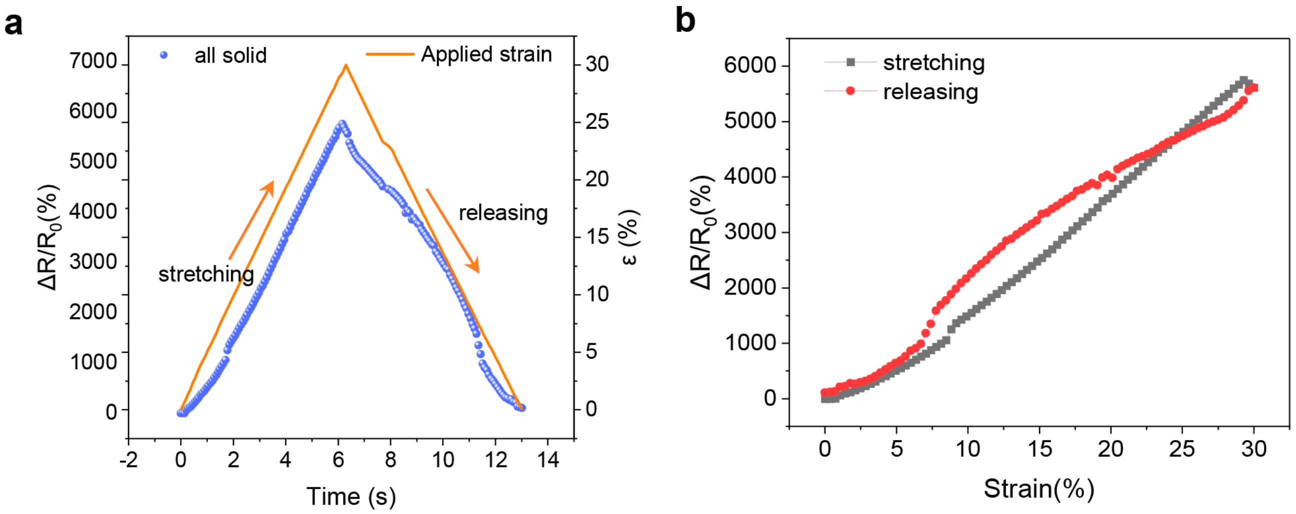

Figure S9. (**a**) Statistic relative resistance change($\Delta R/R_0$) versus applied strain(ε); (**b**) Relative resistance change variation versus applied strain during stretching and releasing.

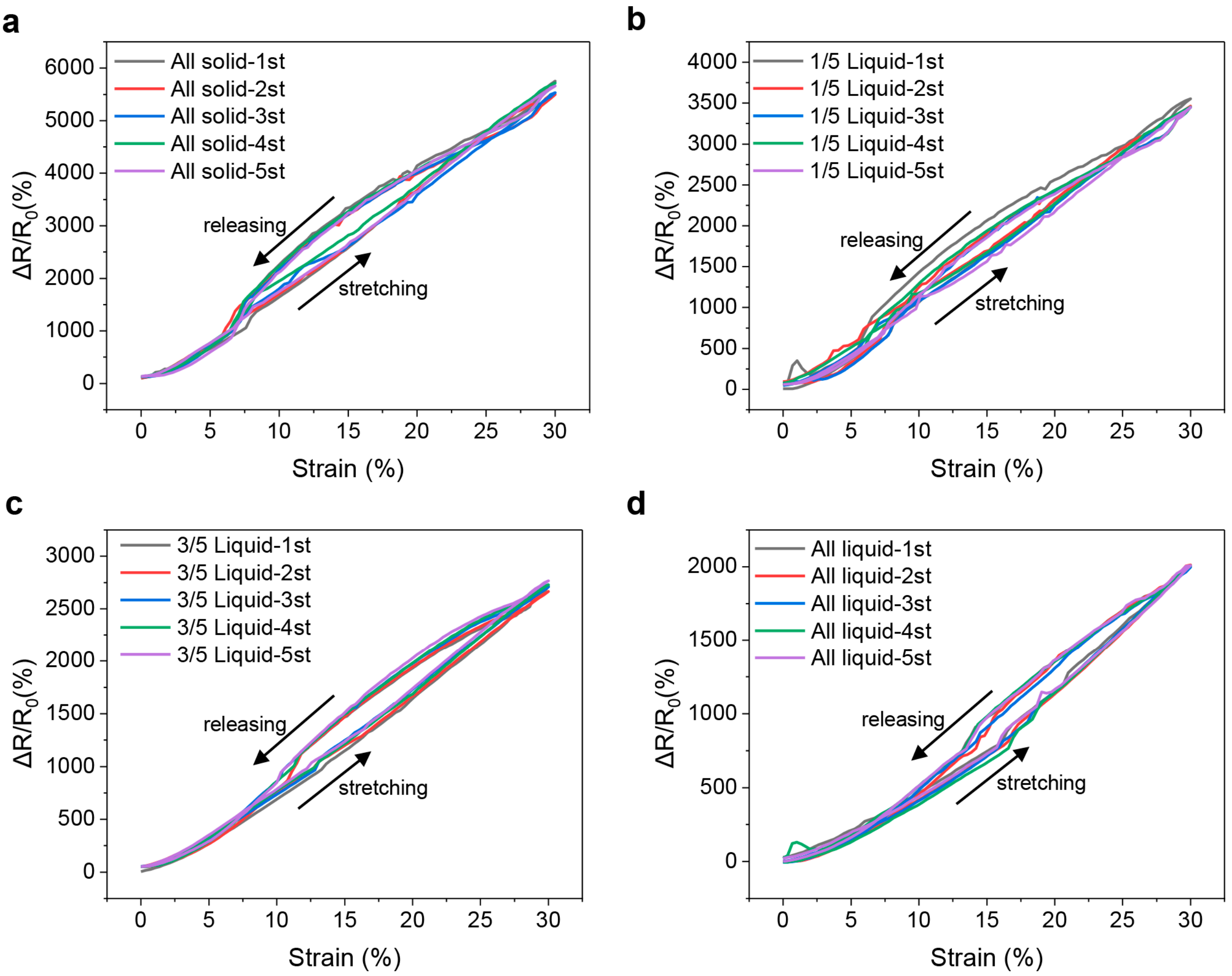

Figure S10. The hysteresis curve of the heterogeneous strain sensor from the cyclic uniaxial tensile test under different states: (**a**) All solid state; (**b**) 1/5 liquid state; (**c**) 3/5 liquid state; (**d**) All liquid state.

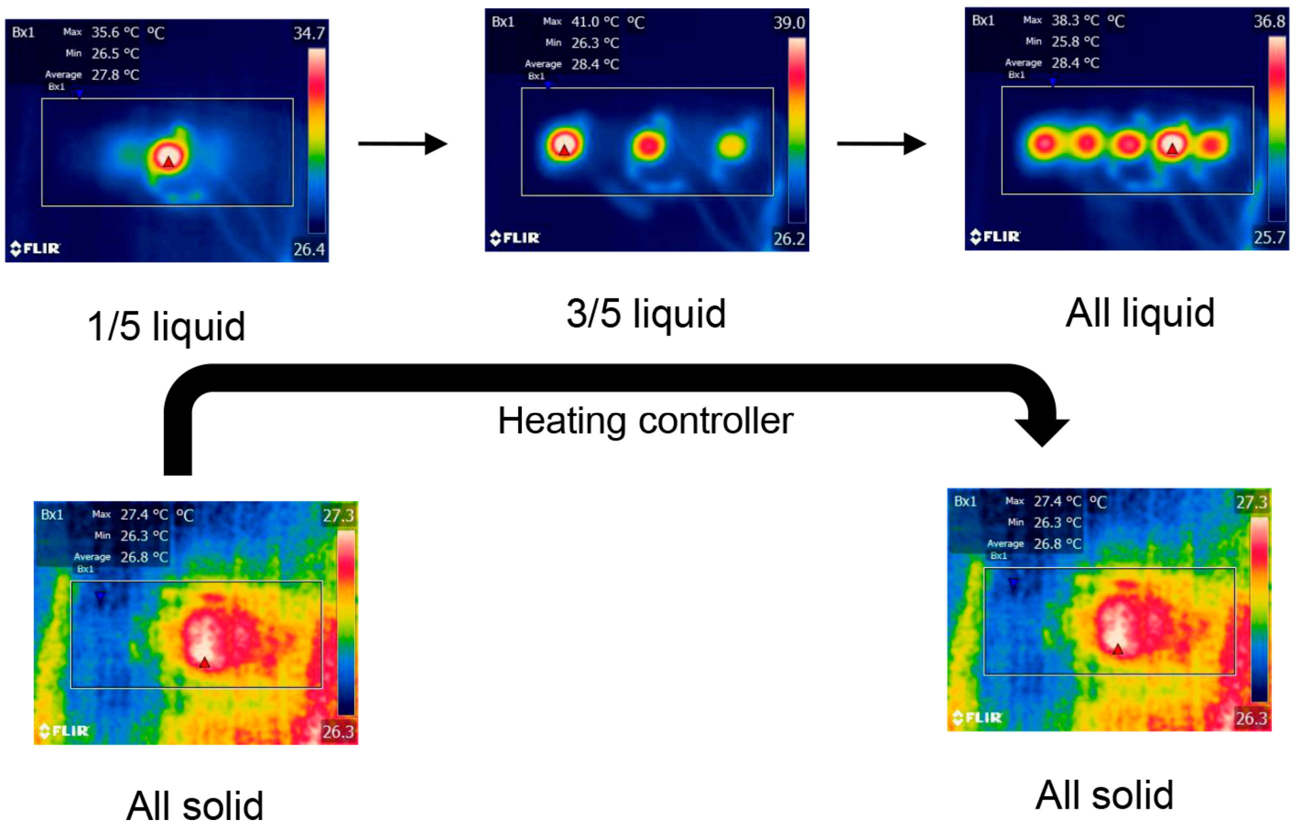

Figure S11. The thermal imaging distribution of the LM cells during the electric regulation process.

Table S1. Performances of resistive-type strain sensors. The sensitivity of conventional strain sensors is always scattered and independent.

Reference	Active materials	Maximum gauge factor	Maximum stretchability (%)
[1]	Graphene/CNT	3	20
[2]	CNT	75.8	50
[3]	CNTs	80	100
[4]	Graphene	88.4	55
[5]	Aligned graphene	10.28	22
[6]	Porous CNTs-GR/PDMS	186.5	120
[7]	Graphene ink	1054	26
[8]	Graphene/PAA	~1	500
This work	Graphene/CNT	90~220	30~45

References

1. Shi, J.; Hu, J.; Dai, Z.; Zhao, W.; Liu, P.; Zhao, L.; Guo, Y.; Yang, T.; Zou, L.; Jiang, K.; et al. Graphene welded carbon nanotube crossbars for biaxial strain sensors. *Carbon* **2017**, *123*, 786-793, doi:10.1016/j.carbon.2017.08.006.
2. Pan, S.; Liu, Z.; Wang, M.; Jiang, Y.; Luo, Y.; Wan, C.; Qi, D.; Wang, C.; Ge, X.; Chen, X. Mechanocombinatorially Screening Sensitivity of Stretchable Strain Sensors. *Adv. Mater.* **2019**, *31*, e1903130, doi:10.1002/adma.201903130.
3. Liu, Z.; Qi, D.; Hu, G.; Wang, H.; Jiang, Y.; Chen, G.; Luo, Y.; Loh, X.J.; Liedberg, B.; Chen, X. Surface Strain Redistribution on Structured Microfibers to Enhance Sensitivity of Fiber-Shaped Stretchable Strain Sensors. *Adv. Mater.* **2018**, *30*, 1704229, doi:10.1002/adma.201704229.
4. Wu, S.; Peng, S.; Han, Z.J.; Zhu, H.; Wang, C.H. Ultrasensitive and Stretchable Strain Sensors Based on Mazelike Vertical Graphene Network. *ACS Appl. Mater. Interfaces* **2018**, *10*, 36312-36322, doi:10.1021/acsami.8b15848.
5. Huang, S.; He, G.; Yang, C.; Wu, J.; Guo, C.; Hang, T.; Li, B.; Yang, C.; Liu, D.; Chen, H.J.; et al. Stretchable Strain Vector Sensor Based on Parallelly Aligned Vertical Graphene. *ACS Appl. Mater. Interfaces* **2019**, *11*, 1294-1302, doi:10.1021/acsami.8b18210.

6. He, Y.; Wu, D.; Zhou, M.; Zheng, Y.; Wang, T.; Lu, C.; Zhang, L.; Liu, H.; Liu, C. Wearable Strain Sensors Based on a Porous Polydimethylsiloxane Hybrid with Carbon Nanotubes and Graphene. *ACS Appl. Mater. Interfaces* **2021**, doi:10.1021/acsami.0c22823.
7. Yang, Y.F.; Tao, L.Q.; Pang, Y.; Tian, H.; Ju, Z.Y.; Wu, X.M.; Yang, Y.; Ren, T.L. An ultrasensitive strain sensor with a wide strain range based on graphene armour scales. *Nanoscale* **2018**, *10*, 11524-11530, doi:10.1039/c8nr02652a.
8. Lin, S.; Zhao, X.; Jiang, X.; Wu, A.; Ding, H.; Zhong, Y.; Li, J.; Pan, J.; Liu, B.; Zhu, H. Highly Stretchable, Adaptable, and Durable Strain Sensing Based on a Bioinspired Dynamically Cross-Linked Graphene/Polymer Composite. *Small* **2019**, *15*, e1900848, doi:10.1002/smll.201900848.
